# Effect of the Water-Binder Ratio on the Autogenous Shrinkage of C50 Mass Concrete Mixed with MgO Expansion Agent

**DOI:** 10.3390/ma16062478

**Published:** 2023-03-21

**Authors:** Jun Chen, Zhongyang Mao, Xiaojun Huang, Min Deng

**Affiliations:** 1 College of Materials Science and Engineering, Nanjing Tech University, Nanjing 211816, China; 202061103064@njtech.edu.cn (J.C.);; 2 State Key Laboratory of Material-Oriented Chemical Engineering, Nanjing Tech University, Nanjing 211800, China

**Keywords:** MgO expansion agent, autogenous shrinkage, C50 mass concrete

## Abstract

The high adiabatic temperature rise and low heat dissipation rate of mass concrete will promote rapid hydration of the cementitious material and rapid consumption of water from the concrete pores, which may significantly accelerate the development of concrete autogenous shrinkage. In this study, the effect of the water-binder ratio on the autogenous shrinkage of C50 concrete mixed with MgO expansion agent (MEA) was explained with respect to mechanical properties, pore structure, degree of hydration, and micromorphology of the concrete based on a variable temperature curing chamber. The results show that the high temperature rise within the mass concrete accelerates the development of early (14 d) autogenous shrinkage of the concrete, and that the smaller the water-binder ratio, the greater the autogenous shrinkage of the concrete. With the addition of 8 wt% MEA, the autogenous shrinkage of concrete can be effectively compensated. The larger the water-binder ratio, the higher the degree of MgO hydration, and in terms of the compensation effect of autogenous shrinkage, the best performance is achieved at a water-binder ratio of 0.36. This study provides a data reference for the determination of the water-binder ratio in similar projects with MEA.

## 1. Introduction

High-strength concrete is being used more widely due to the increasing demands of modern construction [1,2]. Compared with ordinary concrete, high-strength concrete has a lower water-binder ratio (typically ≤ 0.4) due to the addition of high-efficiency water-reducing agents [3,4,5]. When the water-binder ratio of concrete is less than 0.40 [6], autogenous shrinkage accounts for a greater proportion of the total shrinkage, which is an important factor causing concrete cracking [7,8,9,10]. The study of autogenous shrinkage in high-strength concrete needs more attention as it shortens the service life of concrete structures.

A great deal of research has been carried out and solutions have been proposed, both nationally and internationally, on how to reduce the autogenous shrinkage of concrete [11,12,13]. There are currently two mainstream methods. One method is to add lightweight aggregates [14,15,16,17,18], or highly absorbent polymers [19,20] and other internal curing materials to the concrete for internal curing, in order to reduce the rate of self-drying inside the concrete and thus reduce the autogenous shrinkage of high-strength concrete. However, there are few practical engineering applications due to the disadvantages of reducing the strength of concrete and the high cost. The second method is to add an expansion agent to the concrete to compensate for the autogenous shrinkage of the concrete through the expansion effect produced by the expansion agent. There are three main types of concrete expansion agents commonly used in practical engineering: sulfate of alumina, calcium oxide, and magnesium oxide [21,22]. The sulfate of alumina expansive agent requires a large amount of water—in concrete with a low water-binder ratio, its expansion effect cannot be fully achieved. Moreover, its hydration product, calcium alumina, is easy to decompose at high temperatures, and there is a risk of expansion collapsing. Calcium oxide expansion agents’ hydration is too fast, and the effect of shrinkage compensation for aged concrete is weak. On the contrary, magnesium oxide expansion agents have the advantages of low water requirements, stable products, and controlled expansion rates; therefore, they are often used to compensate for the long-term shrinkage of mass concrete [23].

Under normal temperature conditions, the use of MEA to compensate for autogenous shrinkage of concrete has been well documented [24,25]. However, in some large volume straight wall concrete with special applications, the poor thermal conductivity of bulk concrete and the large amount of heat of hydration given off by the hydration of cement can cause the temperature of the structural concrete to rise rapidly, which may significantly accelerate the development of concrete autogenous shrinkage [26,27,28] and affect the use of MEA. Therefore, the effect of temperature must be considered when using MEA to compensate for shrinkage. The effect of maintenance temperature on the expansion of MEA on concrete has been extensively studied [29,30,31,32]. However, MgO has a strong temperature sensitivity [33] and the expansion effect of MEA is influenced by the internal temperature history of bulk concrete, so the performance of MEA cannot be predicted according to a constant temperature. Li et al. [34] investigated the synergistic effect of MEA and internal curing materials on the shrinkage compensation of concrete under variable temperature conditions, relying on urban railway projects. Wang et al. [35] studied the compensating effect of a magnesium oxide expander with different activity and different dosing levels on the shrinkage of panel concrete. Li et al. [36] demonstrated the expansion performance of a magnesium oxide expander with different reactivity in ship lock concrete and combined it with different temperature histories. Ou et al. [22] used a self-developed temperature stress testing machine to study the crack mitigation properties of different reactive magnesium oxide expansion agents in concrete under different engineering conditions. These studies show that the compensatory effect of MEA on shrinkage varies in different projects. The current application of MEA in engineering is mostly focused on its reactivity and the amount of admixture, ignoring the effect of concrete mixing water on the compensatory shrinkage performance of MEA at variable temperatures. At room temperature, the autogenous shrinkage is higher with the reduction of the water-binder ratio [37,38]. Cao et al. [39] proved that the larger the water-binder ratio, the better the compensation effect when using MEA to compensate for concrete shrinkage at room temperature. However, based on the temperature history of actual projects, there are still few reports on whether the effect of temperature on the autogenous shrinkage of different water-binder ratios—or the effect on the expansion of MEA—can have a cross-matched effect, and what kind of water-binder ratio can achieve the best effect. This may lead to the incorrect use of MEA in practical engineering.

This study is based on a large volume straight wall concrete project, using the same concrete mix ratio as that of the straight wall on site (8 wt% of MEA). Through a simulation of the site temperature history (high temperature peak and low cooling rate), the effect of the water-binder ratio on the autogenous shrinkage of concrete with MEA under the variable working temperature conditions was investigated, in order to provide a basis for the selection of the water-binder ratio of concrete for engineering structures. 

## 2. Materials and Methods

### 2.1. Materials

**Figure 1:**
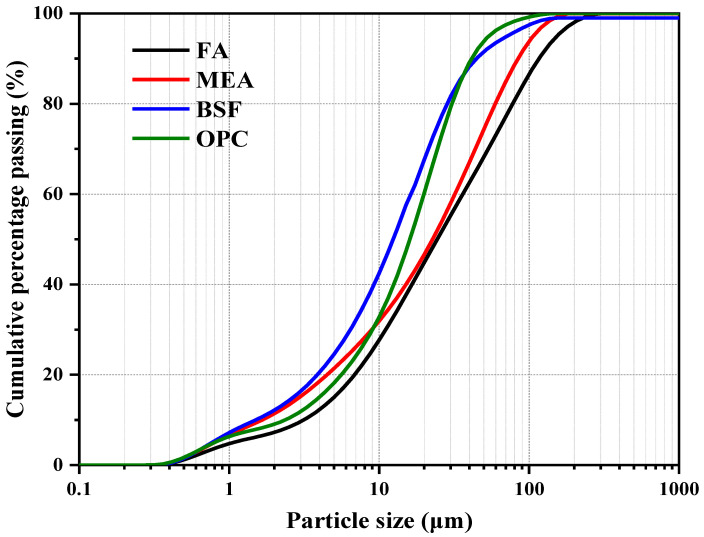
Particle size distribution of raw materials.

In this research, Ordinary Portland cement (OPC) was provided by Jiangnan Onoda Cement Co., Ltd. (Nanjing, Jiangsu Province, China). Fly Ash (FA) was supplied from Nanjing, and blast furnace slag (BFS) was provided by Mabel Ltd. (Nanjing, Jiangsu Province, China), which were used as the supplementary cementitious materials. The MEA was supplied by Nanjing Subote New Material Co., Ltd. (Nanjing, Jiangsu Province, China), of which the reactivity value was 200 s. Its reactivity was estimated by the citric acid method in accordance with Chinese standard CBMF-2017 [40]. The chemical compositions of OPC, FA, BSF, and MEA are provided in Table 1. Distributions of particle size of these cementitious materials are presented in Figure 1. A polycarboxylate superplasticizer (SP), provided by Nanjing Subote New Material Co., Ltd., was used for maintaining a consistent slump of concrete, whose water reduction rate was 30% and solid content was 40%.

### 2.2. Mixture Proportions of Concrete

Four concrete mixtures (numbered from C1 to C4) containing 8 wt% MEA are shown in Table 2, whose water-binder ratios range from 0.28 to 0.40. Mixtures C5–C8 were designed as references, which were mixed without MEA in this research. In mixtures C1–C4, FA, BFS, and MEA were used as a partial replacement for cement in order to reduce the total hydration heat release and the temperature peak of bulk concrete, which may decrease the high risk of concrete cracking. The amount of sand (S) and gravel (G) used is also shown in Table 2, and the sand percentage is 38%. The quantity of MEA is determined according to the actual project, in which the addition of MEA at 8 wt% of the total cementitious materials restrained the cracking of concrete walls, whereas 6 wt% did not. This is also supported by the research of Yu [41].

### 2.3. Methods

#### 2.3.1. Mechanical Properties

**Figure 2:**
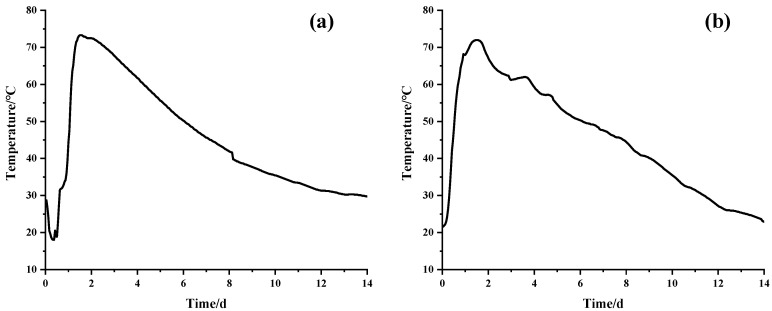
Temperature curves: (**a**) temperature history of straight walls; (**b**) temperature history of the test chamber simulation.

A variable temperature curing chamber was used to simulate the history of changes in the central temperature of straight walls in engineering. Figure 2a presents a typical temperature history profile for large volume straight wall concrete of civil engineering, which has a high temperature rise and low cooling rate. The central temperature of the concrete achieves a temperature peak at around 30 h and drops to room temperature at around 14 days. Figure 2b presents the curve of temperature change simulated in the laboratory.

According to Chinese standard GB/T 50081-2019 [42], the concrete was poured into molds whose dimensions were 150 mm × 150 mm × 150 mm. Cured in the variable temperature oven for 24 h, concrete specimens would be removed from molds and wrapped in plastic wrap, after which they continued to be cured. The compressive strength of concrete was tested at 3 d, 7 d, and 28 d—and an average for each sample, at each age, was taken from triplicate tests.

#### 2.3.2. Autogenous Deformation

**Figure 3:**
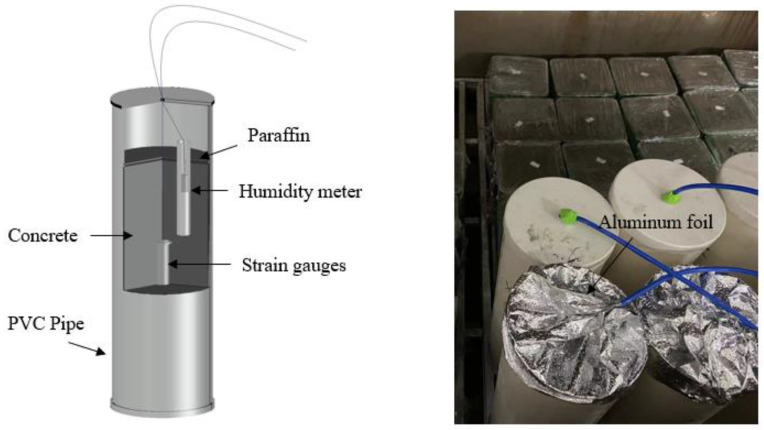
PVC pipe and PVC pipe in a variable temperature curing chamber.

Considering that the concrete of the straight walls in the project was in a sealed state before the early demolding, which would have exacerbated the autogenous drying shrinkage, the autogenous deformation test was performed. The test for autogenous shrinkage of concrete is carried out using the buried strain gauge method with reference to SL/L 352-2020 [43]. After the concrete mixing was completed, fresh concrete was cast into a cylindrical PVC pipe (Ø 160 mm × 400 mm) and a W-15 sine-type strain gauge, as well as a moisture meter, was buried in the middle part of the specimen (Figure 3). Finally, the concrete surface was sealed with paraffin wax. The concrete mixtures loaded in PVC pipes were cured by the variable temperature curing chamber according to the above temperature curve, and the shrinkage was recorded every half hour over 28 days. The final setting time of the concrete was set as the “zero time” for autogenous shrinkage [44].

#### 2.3.3. Thermal Analysis

At 1 d, 3 d, and 7 d, the mixtures of concrete without sand were analyzed to calculate the hydration of magnesium oxide. After being cut into pieces, the samples were soaked in the anhydrous ethanol for a week for the purpose of terminating their hydration. Then, the samples were placed in a vacuum drying oven at 60 °C until the mass was stable. After, those dried samples were ground into powders of no more than 0.080 μm. The powder samples were tested using NETZSCH STA 449F1 thermogravimetry under a N_2_ atmosphere, heated from room temperature to 950 °C.

#### 2.3.4. Pore Structure

The pore structure of concrete specimens was tested using a mercury intrusion porosimeter (MIP). According to the sample preparation requirements, the samples maintained in the variable temperature oven were taken out. Concrete specimens were cut into 3~5 mm pieces and soaked in anhydrous ethanol, which was aimed at terminating the hydration of samples. Afterward, the samples were placed in an oven at the temperature of 60 °C until the mass was stable.

## 3. Results

### 3.1. Effect of Water-Binder Ratio on the Compressive Strength of Concrete with MEA

**Figure 4:**
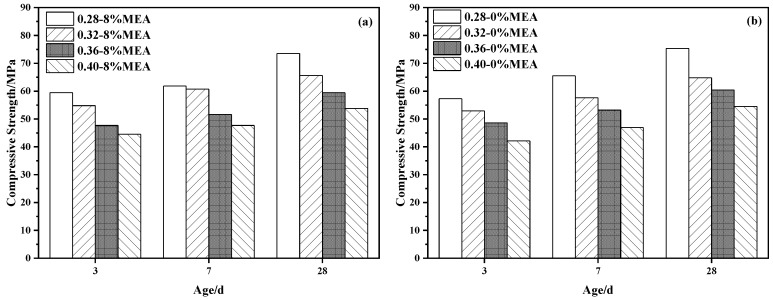
Compressive strength of the concrete with different water-binder ratios: (**a**) with MEA; (**b**) without MEA.

**Figure 5:**
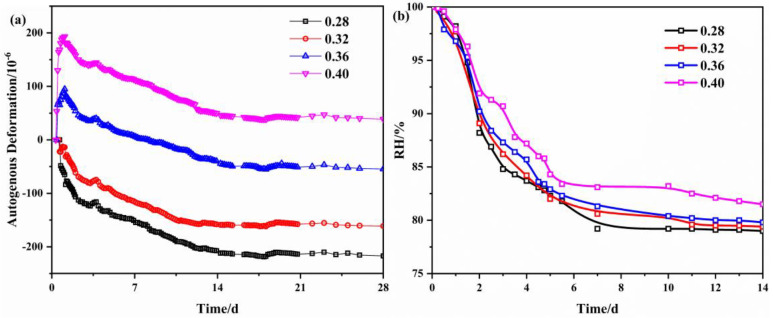

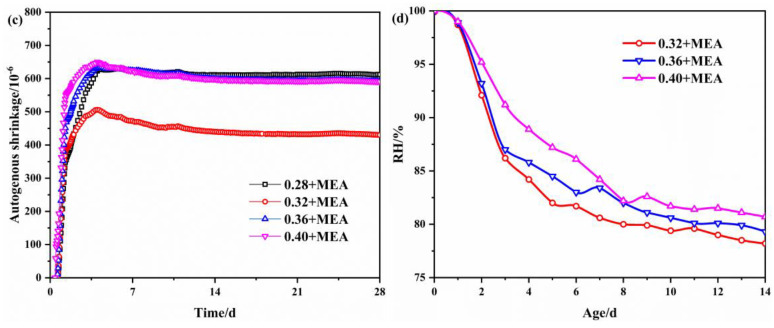
Effect of water-binder ratios on the autogenous deformation and internal relative humidity of concrete: (**a**) autogenous deformation, without MEA; (**b**) internal relative humidity, without MEA; (**c**) autogenous deformation, with MEA; (**d**) internal relative humidity, with MEA.

Figure 4 illustrates the compressive strength of the concrete with different water-binder ratios at different ages. As indicated in Figure 5a, the compressive strength under variable temperature conditions of curing was consistently over 50 MPa at 28 d, and more than 85% of the 28 d strength could be achieved at 7 d. Similar to normal temperature curing, the compressive strength of concrete decreases as the water-binder ratio increases. Compared with the concrete without MEA, which is presented in Figure 4b, MEA hardly affects the compressive strength of concrete. Before 7 d, the compressive strength of the concrete with MEA is slightly lower than that of the concrete without MEA. However, when the concrete is cured for 28 days, both are almost equal in compressive strength. This may be attributed to MEA replacing part of the cement, which lessens the contribution of the hydration products of MgO to strength [45]. In addition, MgO of MEA will compete with cement for water in the early stage, affecting the early hydration of cement; hence, the early strength of concrete mixed with MEA is lower than that of concrete not mixed with MEA. As the reaction proceeds, the slurry structure becomes denser, and the compressive strength of MEA-doped concrete does not differ significantly from that of non-MEA-doped concrete.

### 3.2. Autogenous Deformation and Internal Relative Humidity in Concrete

Figure 5 represents the autogenous deformation of concrete with different water-binder ratios under variable temperatures. As demonstrated in Figure 5a, the autogenous deformation of concrete without MEA shows a tendency toward a smaller water-binder ratio, the larger the autogenous deformation. Concrete whose water-binder ratio is 0.28 had the greatest autogenous deformation at 18 d, up to −218 microstrain. However, when the water-binder ratio was 0.40, the concrete produced 38 microstrain expansion. This confirms the direct link between autogenous deformation of concrete and internal moisture, and it can be concluded that the water-binder ratio of 0.40 is the cut-off at which the issue of autogenous deformation should be taken into account. After 14 d of curing, the autogenous shrinkage tends to steady. The results of the compressive strength of the concrete show that there is little difference between the 7 d compressive strength and the 28 d strength of the concrete. This means that most of the hydration has been completed at 7 d under the simulated temperature history. The self-drying caused by hydration is the main reason for the autogenous shrinkage of the concrete, and therefore, the autogenous shrinkage of the concrete tends to be stable after 14 d. Figure 5c shows the autogenous deformation of concrete with different water-binder ratios after the addition of 8 wt% MEA. As shown in Figure 5c, the autogenous deformation of concrete consistently shows expansion, especially before 7 d, in the period when the autogenous deformation of concrete develops rapidly. After 7 d, it reaches a stable stage of development, and within 28 d, it produces an inverse shrinkage within 100 microstrain. The autogenous deformation of the concrete samples with the water-binder ratios of 0.36 and 0.40 are essentially the same at 28 d, at around 600 microstrain. Compared to the concrete without MEA, that with MEA has a better compensation effect on autogenous shrinkage when the water-binder ratio is 0.36.

Figure 5b,d show the internal humidity curves of concrete for different water-binder ratios. As presented in the subfigures, the addition of MEA has little effect on the internal humidity of concrete. However, it is worth noting that the internal humidity of concrete decreases significantly during 1–7 d, which corresponds to the rapid development of early autogenous shrinkage of concrete. After being cured for 14 d, the humidity slowly decreases, which also explains why the autogenous shrinkage of concrete remains near stable after 14 d. The variation in the internal humidity of the concrete essentially matches the variation of the curing temperature, with high temperatures accelerating the depletion of moisture within the concrete and the development of autogenous deformation.

### 3.3. Thermal Analysis

**Figure 6:**
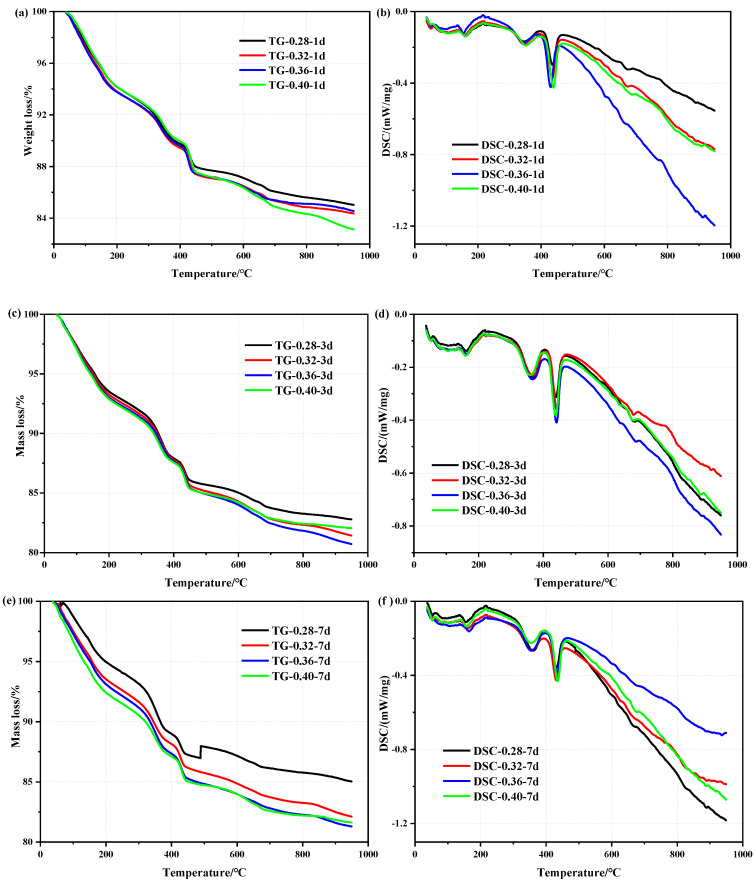
TG/DSC curves of the concrete containing MEA with different water-binder ratios: (**a**) TG curves at the ages of 1 d; (**b**) DSC curves at the ages of 1 d; (**c**) TG curves at the ages of 3 d; (**d**) DSC curves at the ages of 3 d; (**e**) TG curves at the ages of 7 d; (**f**) DSC curves at the ages of 7 d.

Figure 6 shows the TG and DSC profiles of the concrete specimens with different water-binder ratios. As shown in Figure 6b,d,f, there is an obvious endothermic peak that appears at 320–400 °C, which corresponds to the decomposition of Mg(OH)_2_. Based on Mo [46], the quantity of Mg(OH)_2_ and the hydration degree of MgO can be calculated by Equations (1) and (2):

(1) $$Q_{\mathrm{Mg}{\left(\mathrm{OH}\right)}_{2}}=\frac{58\times {\mathrm{Mass}\;\mathrm{loss}}_{\left(320\;℃-400\;℃\right)}}{18}$$

(2) $$H_{\mathrm{MgO}}=\frac{40\times {\mathrm{Mass}\;\mathrm{loss}}_{\left(320\;℃-400\;℃\right)}}{n\times 18\times \left[1-{\mathrm{Mass}\;\mathrm{loss}}_{\left(950\;℃\right)}\right]}$$

Table 3 provides the amount of Mg(OH)_2_ and the hydration degree of MgO in the samples. It is clear that there is a rapid increase in the hydration degree of MgO during the first 3 d. The water-binder ratios (0.28, 0.32, 0.36, 0.40) are 53.1%, 52.3%, 55.2%, and 67.4%, respectively, which are highly dependent on the curing temperature at which the specimens are maintained, once again confirming the temperature sensitivity of the MEA and that high temperatures accelerate the hydration of MgO. The hydration degrees of MgO in concrete with different water-binder ratios are relatively close at 3 d, which is probably attributable to the fact that there is sufficient water inside the concrete in the early stages and MgO competes with cement for water. When at 7 d, there is a notable difference between different water-binder ratios. The hydration degrees of MgO in the concrete with the water-binder ratios of 0.40, 0.36, and 0.40 are 72.6%, 66.9%, and 62.8%, respectively, whereas the hydration degree is only 58.9% when the water-binder ratio is 0.28. Combined with the changes of humidity in Figure 5, the reduction in moisture in the concrete impedes the hydration of MgO. The lower the water-binder ratio, the less free water is available for MgO, or the lower the rate. It is obvious that internal moisture becomes critical in the later stages of curing.

### 3.4. Pore Structure and Microstructure

**Figure 7:**
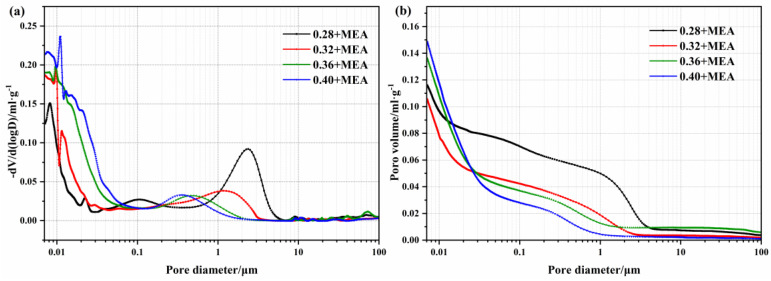
Pore size distributions of the concrete containing MEA with different water–binder ratios at 3 d: (**a**) incremental volume; (**b**) cumulative volume.

**Figure 8:**
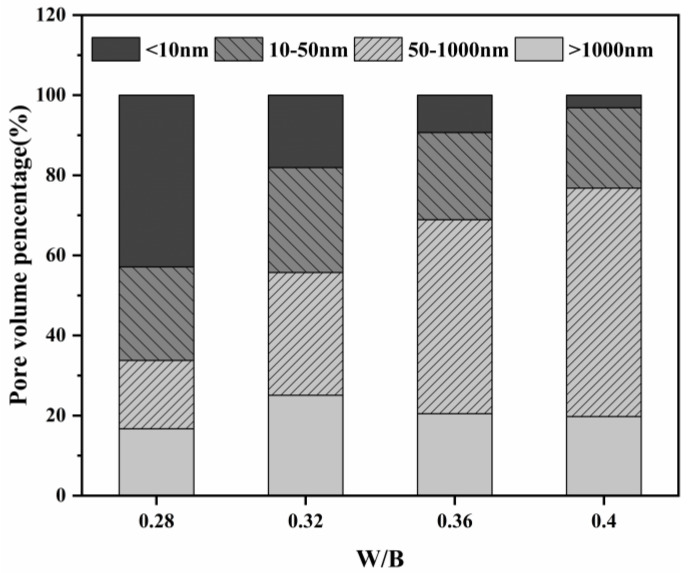
Relative pore volume distribution of concrete pastes.

Figure 7 shows the pore structures of concrete specimens mixed with MEA (8 wt%) and different water-binder ratios at 3 d. As illustrated in Figure 7a, the pore size of the samples added with MEA is mostly distributed in the range of 7~70 nm. As the water-binder ratio increases, the peak of the pore size moves to the right and the pore size < 50 nm increases, probably due to the higher hydration degree of the silicate cement and the formation of more gel pores. Figure 7b indicates that the cumulative porosity of the specimens is also increased. Figure 8 shows the relative pore volume distribution of MgO concrete with various water-binder ratios. It is obvious that the larger the water-binder ratio, the fewer pores there are above >50 nm. The increase in water contributes to the migration of Mg^+^, which allows more Mg(OH)_2_ products to grow inside the pores, thus changing the pore size distribution inside the pastes and refining the pore size.

**Figure 9:**
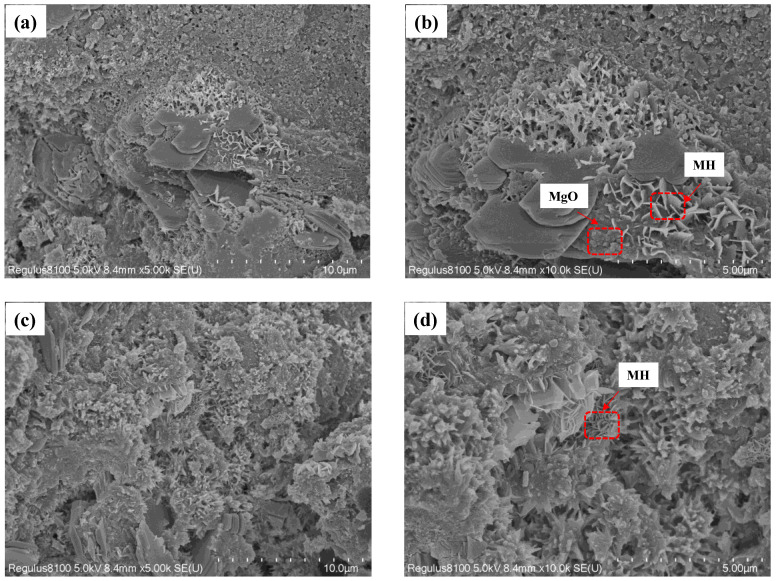
SEM images of hydrated MgO in concrete pastes at 7 days: (**a**) w/c = 0.32, ×5000; (**b**) w/c = 0.32, ×10,000; (**c**) w/c = 0.36, ×5000; (**d**) w/c = 0.36, ×10,000.

Figure 9 presents the microscopic morphology of the specimens with the water-binder ratios of 0.32 and 0.36. As illustrated in Figure 9b, under high temperature maintenance, the Mg(OH)_2_ crystals are mainly in the form of flakes of different sizes, which grow near the MgO particles. Ca^2+^ has an inhibitory effect on the diffusion of Mg^2+^ and promotes the growth of Mg(OH)_2_; therefore, Mg(OH)_2_ crystals generally grow near Ca(OH)_2_ crystals. The lamellar Mg (OH)2 crystals in Figure 9d are denser and fill in the pores, which explains the smaller pore size of the specimen with a water-binder ratio of 0.36 compared to that with a ratio of 0.28.

## 4. Discussion

Bulk high-strength concrete generally has a high adiabatic temperature rise due to the great amount of cementitious material and the slow dissipation of heat from the concrete. In large volume straight wall concrete projects, temperatures in the center of the concrete can reach over 70 °C (and even 80 °C) and do not drop to room temperature until 14 d. The high temperature changes within the bulk straight wall concrete not only accelerate the development of autogenous concrete deformation but also promote the hydration of MgO. Although the addition of MEA mitigates the autogenous shrinkage of the concrete, the effect of different water-binder ratios on the deformation of concrete under variable temperature conditions varies slightly. Compared to concrete with a water-binder ratio of 0.28, an increased water-binder ratio not only reduces the autogenous shrinkage of the concrete itself but also contributes more to the hydration of MgO and the production of more Mg (OH)_2_. The thermal analysis shows that there is little difference in the degree of hydration of MgO in concrete with different water-binder ratios at 3 d. However, at 7 d, the hydration degree of MgO in concrete with a water-binder ratio of 0.40 is 72.6% compared to 58.9% with a water-binder ratio of 0.28. Sufficient water is supplied to MgO to produce Mg (OH)_2_, which counteracts the pore pressure and is the key to compensating the autogenous shrinkage of concrete. Meanwhile, Ca^2+^ has an inhibitory effect on the diffusion of Mg^2+^, which promotes the nucleation and growth of Mg (OH)_2_. Thus, Mg (OH)_2_ crystals mostly grow overlappingly in pores not far from the MgO particles. Moreover, the higher the water-binder ratio, the denser the hydration product of MgO. The early arrival of the hydration period of MgO allows the crystalline growth pressure of the Mg (OH)_2_ crystals to resist the early shrinkage deformation of the concrete. However, the autogenous deformation of mass concrete in practical projects is more complex, as the restraint of reinforcement, the large temperature difference between day and night, and the method of construction all affect the autogenous shrinkage of concrete. Increasing or changing the water-binder ratio alone will not completely overcome the problem of autogenous deformation of concrete and needs to be carried out in cooperation with other effective measures according to the actual situation of the project at the time. This study was carried out based on a large volume of straight wall concrete and is intended to provide a reference for similar projects with MEA.

## 5. Conclusions

The internal temperature variations of C50 bulk straight wall concrete structures in practical projects were simulated in the laboratory. On this basis, the influence of different water-binder ratios on the compressive strength, autogenous shrinkage, pore size, and micromorphology of concrete with MEA was studied. The key conclusions are the following:

1. The hydration process of MgO is accelerated under variable temperature curing. The addition of 8 wt% MEA reduces the compressive strength of the concrete, but the effect is minimal (no more than 5 MPa). In this experiment, the compressive strength of the concrete at 7 d can reach 85% or more at 28 d.
2. High temperature accelerates the early self-shrinkage of concrete, especially within 7 d. Increasing the water-binder ratio can reduce the autogenous shrinkage of concrete. When the water-binder ratio is 0.40, the concrete deformation is slightly expanded. Compared to the reference group without MEA, the best effect of compensating for autogenous shrinkage is achieved by adding 8% MEA with a water-binder ratio of 0.36. Therefore, in actual projects, the water-binder ratio can be relaxed to 0.36 or even 0.40 under the premise of ensuring strength.
3. At high temperatures, the hydration product of MgO, Mg(OH)_2_ crystals, appear as lamellar in the cement system, growing mainly in the pores not far from the MgO particles and overlapping each other. As the water-binder ratio increases, the MgO hydrates more and the Mg(OH)_2_ crystals become denser, which fills the pores better and counteracts the autogenous shrinkage of the concrete.

## Figures and Tables

**Table 1:** Chemical compositions of raw materials.

Chemical Compositions	OPC	FA	BFS	MEA
CaO (%)	65.32	4.07	38.00	1.98
SiO_2_ (%)	18.55	50.53	33.72	3.87
Al_2_O_3_ (%)	3.95	31.65	17.74	1.03
Fe_2_O_3_ (%)	3.41	4.48	0.77	0.88
MgO (%)	1.01	0.92	6.35	89.37
K_2_O (%)	0.72	1.26	0.41	0.08
Na_2_O (%)	0.18	0.68	0.41	-
SO_3_ (%)	2.78	1.32	1.04	0.06
LOI (%)	2.88	2.77	−0.72	2.38

**Table 2:** Mixture ratio of concrete (kg/m^3^).

Sample	W/B	Mix Proportion/(kg/m^3^)						
		OPC	FA	BFS	S	G	MEA	SP
C1	0.28	360	90	40	680	1100	40	3.3%
C2	0.32	360	90	40	680	1100	40	2.6%
C3	0.36	360	90	40	680	1100	40	1.9%
C4	0.40	360	90	40	680	1100	40	1.6%
C5	0.28	320	90	40	680	1100	0	3.2%
C6	0.32	320	90	40	680	1100	0	2.5%
C7	0.36	320	90	40	680	1100	0	1.7%
C8	0.40	320	90	40	680	1100	0	1.4%

**Table 3:** Quantity of Mg(OH)_2_ and hydration degree of MgO in concrete pastes containing MEA.

	0.28 + 8% MEA			0.32 + 8% MEA			0.36 + 8% MEA			0.40 + 8% MEA		
	1 d	3 d	7 d	1 d	3 d	7 d	1 d	3 d	7 d	1 d	3 d	7 d
Mass loss at 320–400 °C/wt%	2.1	2.8	3.1	2.2	2.9	3.2	2.2	3.2	3.3	2.2	3.6	3.4
Quantity of Mg(OH)_2_/%	6.8	9.3	10.0	7.2	9.5	10.4	8.2	10.5	10.7	7.2	11.7	11.1
Hydration degree of MgO/%	39.2	53.1	58.9	40.5	52.3	65.8	42.7	55.2	66.9	51.7	67.4	72.6

## Data Availability

The data presented in this study are available on request from the corresponding author.
